# Exchanging missives and missiles: the roles of extracellular vesicles in plant–pathogen interactions

**DOI:** 10.1093/jxb/erx369

**Published:** 2017-11-28

**Authors:** Petra C Boevink

**Affiliations:** The James Hutton Institute, Invergowrie, Dundee, UK

**Keywords:** Antifungal, apoplast, exosomes, extracellular vesicles (EVs), fungal growth, fungal spores, growth inhibition, intercellular communication, plant defence, proteomic analysis

## Abstract

This article comments on:

**Regente M, Pinedo M, San Clemente H, Balliau T, Jamet E, de la Canal L.** 2017. Plant extracellular vesicles are incorporated by a fungal pathogen and inhibit its growth. Journal of Experimental Botany **68,** 5485–5495.

**Extracellular vesicles (EVs) are secreted by organisms from all forms of life. In the mammalian field they are intensively studied due to their importance in disease and potential for therapeutic use. However, there has been little research in plants and thus the paper by** Regente *et al.* (2017 **) is a valuable addition to a small but hopefully growing body of data. The authors conducted proteomic analysis on purified sunflower EVs and demonstrated that they are enriched in defence-related proteins. They found that fungal spores treated with fresh EV preparations are damaged and show reduced growth.**

In addition to being a means to unconventionally secrete proteins to the apoplast, extracellular vesicles (EVs) are presumably being secreted by plant cells for communication with neighbouring plant cells and for interaction with microbes and other organisms. Indeed, it is conceivable that the latter might be more important as it could be argued that plasmodesmata can take care of the bulk of protein and RNA exchange between neighbouring plant cells. On the other hand biology loves complexity and redundancy.

There are several potential pathways for the production of EVs and different classes of EVs are recognized, such as microvesicles and exosomes (Mulcahy *et al.*, 2014). Exosomes are described in the mammalian literature as originating from multivesicular bodies (MVBs). Some of the potential pathways for EV production and uptake are outlined in Box 1. However, another potential source has been identified in plants: the exocyst-positive organelle (EXPO; Wang *et al.*, 2010), which is probably equivalent to specialized secretory autophagosomes identified in yeast (Bruns *et al.*, 2011). In the Arabidopsis EV proteome published by Rutter and Innes (2017), the RPM1 INTERACTING PROTEIN 4 (RIN4) was detected and this has been shown to recruit an exocyst subunit EXO70B1 to the plasma membrane (Sabol *et al.*, 2017). Does a plant cell simultaneously secrete different types of EVs that have distinct target cells, such as other plant cells and microbes? Do different cargos occur in distinct EV types (i.e. that the cargo of exosomes is distinct from that of microvesicles but all exosomes are much the same) or can the same type show variety? If the latter, then sorting mechanisms for cargo selection paired with different EV-targeting components would be required. A scenario develops for an EV-generating system every bit as complex as any other part of the endomembrane system.

If there are different populations within the classes of EV how could they be separated to study their content? Proteomic data should reveal EV membrane markers that may allow distinct classes of EV to be purified for detailed study of their protein cargo.

From the point of view of plant–pathogen interactions, interest is focused on exosomes being produced by the protagonists that are targeted to each other’s cells. What determines the specificity and direction of travel during EV exchange? It would be inefficient if a source cell reabsorbed its own EVs and potentially dangerous for a plant cell to absorb EVs containing damaging molecules or enzymes that have been secreted to attack pathogen cells. The most straightforward mechanism for generating specificity would be to decorate the EV membranes with targeting proteins, glycoproteins or other molecules creating a system similar to the mechanisms of vesicle targeting within the cell. The targeting proteins would interact with proteins or other identifiers on the target cells and perhaps even stimulate uptake. Human cancer cell exosome uptake was reduced to 45% after the exosomes had been treated with proteinase K, and treatment of the cells with proteinase K also reduced exosome uptake, by 32% (Escrevente *et al.*, 2011). Uptake was also reduced when cells were treated with an excess of monosaccharides, indicating the importance of glycoprotein recognition. Regente *et al.* (2017) identified mannose-binding lectins in the EV proteome, which are well known to be involved in pathogen recognition and plant defence (Lannoo and Van Damme, 2014) and in mammalian systems have been shown to stimulate uptake by immune cells (e.g. Jack *et al.*, 2005). The EV proteomes generated by both Regente *et al.* (2017) and Rutter and Innes (2017) also included LRR-containing proteins that are characteristic of defence receptors.

We should not forget that some, or perhaps many, exosomes are thought simply to degrade in the apoplast to allow the release of cytoplasmic proteins through unconventional secretion. Are there specific EV populations that are differentially fated – to either degrade or fuse with the target cell? Does the intimate plant–pathogen or plant–symbiont interface zone have specific properties that favour uptake over degradation?

**Box 1. Potential routes out and in for extracellular vesicles**

For conventional secretion (1) proteins enter the endoplasmic reticulum (ER) co-translationally then pass through the Golgi body and are packaged into transport vesicles that fuse with the plasma membrane. For unconventional secretion several routes have been proposed. Classically, unconventional secretion describes how proteins without signal peptides, which are presumably therefore synthesized in the cytoplasm (black stars), are secreted into the extracellular milieu (the apoplast in the case of plants). This may be via invagination into multivesicular bodies [MVB, an organelle that is regarded as evolving from the trans-Golgi network (TGN)] (2) or direct budding from the plasma membrane (3) followed by degradation of the vesicle membrane in the apoplast (4). The vesicles that emerge via these routes have an alternative fate, however, which is to stay as extracellular vesicles (EVs) and be taken up by another cell. It is possible that some of them fuse directly with the target cell’s plasma membrane (5) but the evidence favours endocytosis of the majority. The endocytosis may be preceded by recognition of the vesicle components by receptors on the target cell (blue cylinder; 6) or by recognition of proteins, carbohydrates or lipids on the target cell by components of the vesicle membrane (zigzag; 7). Once endocytosed the vesicle contents could be released by degradation of the vesicular membranes (8) or through retrograde trafficking to the late endosome (orange arrows), which is largely equivalent to the MVB, and fusion of the vesicular membrane with the bounding membrane (9).

The picture is complicated by unconventionally secreted proteins that do have signal peptides (red stars). It is not difficult to understand how they could end up within the bounding membrane of the exosome positive organelle (EXPO), which is probably a form of autophagosome and may thus form from a cup-shaped ER extension that engulfs cytoplasm and potentially organelles. It is also possible that there are transient connections between the ER and MVBs (10) by which means the protein could enter the MVB without passing through the Golgi apparatus. These proteins would then be released into the apoplast or could perhaps associate with the exterior of EVs and make their way inside the target cells then to be released by vesicle degradation, or possibly by trafficking all the way to the target cell ER and hijacking the unfolded protein response pathway to exit from the ER (11). It is more difficult to envisage whether there could also be routes by which these proteins end up inside EVs (question marks).

**Figure:**
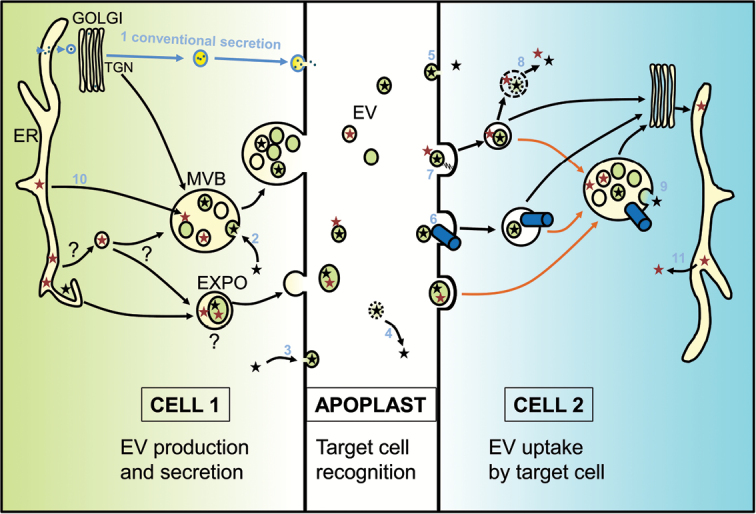

## Plant EVs on the attack

In Regente *et al.* (2017) fungal spores showed signs of membrane disruption after exposure to fresh sunflower EV preparations by uptake of propidium iodide and Evans Blue dyes and some apparent rupture. Following exposure to FM4-64-labelled EVs, the FM4-64 dye accumulated inside fungal spores rather than at the spore plasma membrane. This indicates that the EVs are being endocytosed as opposed to fusing with the spore plasma membrane. Regente *et al.* (2017) tested artificial vesicles and found no effect on spore vitality but at this stage it is difficult to guess how equivalent they were to EVs. Labelling of the artificial vesicles with FM4-64 would show whether they were taken up by the spores. If EVs interact with target membranes through proteins or other molecules on their surfaces as for mammalian exosomes (Escrevente *et al.*, 2011), and this interaction is required for uptake, then the artificial vesicles may not interact appropriately for uptake. Treatment of plant EVs with proteases to remove surface proteins as has been done for mammalian exosomes would indicate whether the system in plants also depends on surface proteins.

The treatment of fungal spores by Regente *et al.* (2017) with a concentrated preparation of EVs was likely to be beyond anything the spores would experience in nature. However, at the point of contact between pathogen and host, at haustoria for example, the local concentration of EVs may be quite high. If spore disruption was not caused by direct membrane effects then it must be a result of the actions of the proteins or compounds that the EVs were carrying. The exosome proteomes described by Rutter and Innes (2017) and Regente *et al.* (2017) were rich in defence proteins including those involved in the myrosinase–glucosinilate system, which produces toxic compounds.

Just as EVs have great potential as therapeutic agents in the medical field, the characterization of plant EVs that target pathogens could lead to novel crop protection strategies. If the proteins and glycoproteins present on pathogen-targeting EVs are found to be specific this could potentially allow the production (either enhanced *in planta* or in a culture system) of EVs carrying highly effective biocides or RNAs that are specifically taken up by economically important pathogens or pests. Host-induced gene silencing is a method shown to provide crop protection (Koch *et al.*, 2013) that is almost certainly dependent on EV delivery of RNA.

## The fight back

In addition to plants producing EVs to attack potential pathogens the reverse is also likely – that pathogens produce EVs to control and attack plants. Pathogens, symbionts and pests have all been shown to produce effectors to control the responses of their host plants. Some of these effectors have been shown to enter host cells and many others are assumed to do so; these are classed as cytoplasmic effectors (as opposed to apoplastic effectors, which function outside the host cell). Bacterial pathogens have specific structures such as the type 3 secretion system to deliver their effectors but no such structures have been identified for eukaryotic pathogens. Giraldo *et al.* (2013) demonstrated that cytoplasmic effectors are non-conventionally secreted by *Magnaporthe oryzae* and we recently showed that an oomycete RXLR effector was secreted through an unconventional route (Wang *et al.*, 2017). These studies open up the possibility that these effectors are secreted in association with EVs. Can we detect effectors in EV preparations? Are pathogen-derived EVs endocytosed by host cells? How are they specifically targeted to host cells? How do cytoplasmic effectors reach their ultimate destinations within host cells? This is currently a hot topic in plant pathology. One curious feature to note is that the RXLR effector Pi04314, and indeed all characterized RXLR class effectors, possess signal peptides. Exosomes are described as being generated by invagination of the MVB membrane which results in engulfment of cytoplasmic content. Microvesicles are described as budding directly from the plasma membrane, and thus also engulf cytoplasmic content. Signal peptide-containing proteins, however, will have entered the endoplasmic reticulum co-translationally, so what is their route to unconventional secretion? The review by Ding *et al.* (2012) summarizes evidence for unconventional secretion of both cytoplasmic proteins and ER contents. They include in their diagrams how autophagosomes and the related EXPOs may be derived from the ER. Further evidence for this is summarized by Zhuang *et al.* (2016). This would presumably result in signal peptide-positive effectors being associated with the exterior of EXPO-derived exosomes, unless there is invagination of the interior of the two membranes or engulfment of ER-derived vesicles by the developing EXPO.

A further exciting area of study will be characterization of RNA populations in pathogen- and plant-derived EVs. The delivery of small RNAs to control gene expression within the host or mRNAs to ensure the production by the host of specific proteins helpful to the establishment of infection are both possible through EVs as they can protect the vulnerable RNAs from the apoplastic environment. *Botrytis cinerea* has been shown to produce RNAs that are destined for host cells (Weiberg *et al.*, 2013). Rutter and Innes (2017) also cite mRNA movement from the parasitic plant *Cuscuta* to host cells but plasmodesmata are formed between *Cuscuta* and their hosts. Thus mRNAs may move symplastically, though of course this does not rule out an exchange of EVs between *Cuscuta* and its host.

## Conclusions

The proteomics of plant EVs has only been published by Rutter and Innes (2017) and Regente *et al.* (2017). Although it is early days and robust proteomics requires large amounts of repetition, careful and consistent data processing and high-quality reference genomes, results to date provide some logical candidate components, as mentioned above. There is considerable scope for proteomics of plant EV populations produced in response to different conditions and stimuli and from a range of species. Proteomics of trypsin-treated EVs would allow us to see which proteins are associated with the exterior of the EVs.

The analysis of EVs involved in plant–pathogen interactions is only just beginning but their potential for increasing our understanding of the exchanges determining disease outcomes and the potential for the development of new strategies to combat disease in economically important crops will ensure the rapid expansion of this field.
